# Toxicity of sodium nitrite-based vertebrate pesticides for European starlings (*Sturnus vulgaris*)

**DOI:** 10.1371/journal.pone.0246277

**Published:** 2021-03-05

**Authors:** Scott J. Werner, Shelagh T. DeLiberto, Hailey E. McLean, Katherine E. Horak, Kurt C. VerCauteren

**Affiliations:** United States Department of Agriculture, Animal and Plant Health Inspection Service, Wildlife Services, National Wildlife Research Center, Fort Collins, Colorado, United States of America; University of Southern Queensland, AUSTRALIA

## Abstract

In the 21st century, invasive animals rank second only to habitat destruction as the greatest threat to global biodiversity. Socially-acceptable and cost-effective strategies are needed to reduce the negative economic and environmental impacts of invasive animals. We investigated the potential for sodium nitrite (SN; CAS 7632-00-0) to serve as an avian toxicant for European starlings (*Sturnus vulgaris* L.). We also assessed the non-target hazard of an experimental formulation of SN that is being developed as a toxicant for invasive wild pigs (*Sus scrofa* L.). In gavage experiments with European starlings, we identified a lowest observed adverse effect level (LOAEL) for mortality of 2.40% technical SN (w/v; 120 mg SN/kg body mass) and a no observed adverse effect level (NOAEL) for mortality of 1.30% technical SN (65 mg/kg). The exposure of ten starlings to the experimental formulation of SN (10% SN pig toxicant) resulted in one starling mortality during four days of exposure to the toxic bait. Sodium nitrite toxicity presented a moderate hazard to European starlings; thus, the future development of SN as an avian toxicant is dependent upon its cost-effectiveness. We discuss the management of toxic effects and non-target hazards of SN for wild birds, including best practices for toxic baiting of vertebrate pests and management of invasive wild pigs.

## Introduction

Toxic baits or pesticides, along with other strategies, can be efficacious and cost-effective control methods for vertebrate pests and invasive species. Toxic baits have varying modes of action; for example, some affect the nervous system (bromethalin) [1], heart function (vitamin D3) [2], or metabolic processes (phosphine) [3]. Other toxic baits decrease the coagulation of blood (e.g. warfarin, brodifacoum) [4]. In addition to the appropriate mode of action, non-target risk should also be considered when selecting a toxic bait.

The non-target risk of toxic baits should be considered for humans, pets, livestock and non-target wildlife. Toxic baits can harm non-target animals through direct ingestion (primary toxicity) or indirectly through the consumption of other animals that ingested the toxicant (secondary toxicity). Several studies have documented the consumption of both toxic and non-toxic baits by non-target wildlife, including native rodents [5, 6], reptiles [7], and birds [8, 9]. During concentrated rodent eradication efforts in Spain, Sanchez-Barbudo et al. [10] found that granivorous birds had a higher occurrence of primary exposure to anticoagulant rodenticides (51%) than the 259 other species tested. In Australian field efficacy trials of sodium nitrite (SN) bait for the control of invasive wild pigs (*Sus scrofa* L.; feral swine), one domestic cow and four common birds were found dead as a result of direct consumption of toxic baits [11]. The assessment of potential non-target risks due to primary bait ingestion requires knowledge of the species-specific sensitivity of non-target animals to the toxicant and the likelihood that those non-target animals will access, ingest and receive a lethal dose. Additionally, the bait location and the duration of toxic bait exposure may need to be considered [12].

Four critical factors regarding whether toxicant residues in target animals pose a potential secondary hazard to non-target species include: 1) the chemical and toxicological properties of the chemical compound and the formulation, 2) the composition of the bait and how it is applied, 3) the behavior of non-target animals, and 4) local environmental conditions [13]. Many publications characterize the non-target risk of anticoagulant rodenticides. For example, bromadiolone, an anticoagulant rodenticide compound, affects foxes (*Vulpes vulpes* L.) and buzzards (*Buteo buteo* L.) through the consumption of contaminated prey [14]. Similarly, brodifacoum poisoning due to the ingestion of contaminated slugs is a risk for common shrews *(Sorex araneus* L.), European hedgehogs (*Erinaceus europaeus* L.), and European starlings (*Sturnus vulgaris* L.; hereafter starling) [15]. Conversely, Shapiro et al. [16] determined there was "no, or very low risk" of secondary poisoning from SN baits to cats (*Felis catus* L.), dogs (*Canis lupus familiaris* L.), and chickens from the consumption of poisoned brushtail possum (*Trichosurus vulpecula* Kerr) carcasses.

While there are few toxicants registered by the U.S. Environmental Protection Agency (EPA) to control pest rodents and pest birds, there are even fewer toxicants available to control other vertebrate pests in the United States (US). Invasive wild pigs cause considerable damage to the environment, agriculture, and personal property, and they can transmit diseases to other wildlife, livestock, and people. Concerns of humaneness and non-target exposure have limited the use of toxic baits to control invasive wild pigs. New, more effective, and ecologically-sound active ingredients are needed to manage this highly destructive species. Efforts are currently underway to develop SN as the active ingredient of a novel toxic bait called HOGGONE® in Australia and the US. New Zealand has already registered this pig toxicant [17]. As an inorganic salt, SN is a common industrial chemical used to treat textiles, produce dyes, and cure meats [18]. The SN in HOGGONE bait is micro-encapsulated before it is added into a bait matrix of peanut paste and crushed-grain bait [19]. HOGGONE is considered an acceptable and relatively humane toxicant because SN causes acute methemoglobinemia, which results in a quick loss of consciousness through brain hypoxia and death from tissue hypoxia [20, 21]. Sodium nitrite also readily degrades into non-toxic compounds when exposed to air and moisture, and SN does not bioaccumulate in the tissues of animals, thus reducing the risk of secondary poisoning [11]. Nevertheless, there is a need for research to examine the toxicity of SN and Hoggone in non-target birds, especially due to the potential for exposure of birds to the toxic bait during wild pig baiting programs. More specifically, there are no published data regarding the primary toxicity of SN in US bird species.

We conducted three experiments to evaluate the impact of SN-based liquid toxicants in starlings and one experiment to assess the non-target hazard of a SN-based vertebrate toxic bait (i.e., wild pig toxicant) in starlings. The first two experiments compared formulations made with technical SN (hereafter TSN; CAS 7632-00-0, Fisher Scientific, 99.3% pure, technical product) or nanoencapsulated SN (hereafter NESN; CAS 7632-00-0, AquaPower, LLC, American Fork, UT, proprietary product). The micro- and nanoencapsulation of toxicants or pesticides can stabilize reactive molecules and minimize their detection by target animals resulting in increased palatability [19, 22]. In addition, the encapsulation of an active ingredient can increase the bio-availability of chemical compounds in both target and non-target animals [23]. We comparatively tested TSN and NESN to evaluate the effect of nanoencapsulation on the toxicity of SN in starlings. We further tested TSN in Experiment 3 and the experimental wild pig toxicant (i.e., HOGGONE; 10% SN) in Experiment 4.

The objectives of the first three experiments were to determine the effectiveness of SN as a starling toxicant by 1) determining the lowest observed adverse effect level (LOAEL) and the no observed adverse effect level (NOAEL) for mortality of TSN and NESN, 2) investigating the toxicity and acceptability of TSN and NESN in a free-choice experiment, and 3) investigating the free-choice toxicity of TSN at less than LOAEL concentrations. We measured consumption and observed toxicity of the experimental wild pig toxicant (0% and 10% SN) among starlings in Experiment 4.

## Materials and methods

### Ethics statement

We followed all applicable institutional guidelines and ethical standards for the care and use of animals per the NWRC Institutional Animal Care and Use Committee (NWRC IACUC). The NWRC IACUC approved our study protocols (QA-2212, 2346; S.J. Werner-Study Director, and 2871; S.T. DeLiberto-Study Director) before the initiation of this study. Per the NWRC IACUC, all staff participating in these studies completed Animal Care and Use training through the Collaborative Institutional Training Initiative. Colorado Parks and Wildlife approved our Scientific Collection Licenses (license numbers 12TRb2006 and 18TRb2006) for the live-capture of starlings.

### Test subjects and facilities

We used starlings as a model bird species instead of an endemic bird species due to their local overabundance and status as an established invasive species in the US. Additionally, starlings are opportunistic omnivores that are often cohabitant with wild pigs and are thus likely to encounter SN used in wild pig control programs. We minimized the number of animals used to estimate the acute oral toxicity of SN based upon the Organisation for Economic Co-operation and Development [24] guidelines for testing chemicals and EPA suggestions.

We conducted our four experiments at the United States Department of Agriculture’s National Wildlife Research Center (NWRC) in Fort Collins, Colorado, USA. We live-captured 123 starlings (males and females) at dairies and feedlots that were known to suffer feed loss attributable to starlings in northern Colorado. Starlings were live-captured using traps and mist nets and quarantined within group cages (4.9 × 2.4 × 2.4 m; 40–50 birds per cage) in the NWRC outdoor animal research facility for at least five days prior to testing. Starlings were acclimated within individual test cages (61 × 45 × 42 cm) for Experiments 1–3, and group cages (3.6 x 1.8 x 2.1 m) for Experiment 4, in the NWRC Invasive Species Research Building for 3–5 days prior to each experiment. Indoor testing conditions included 12 hours of light (0600–1800 hrs) for Experiments 1–3 and 10.5 hours of light (0700–1730 hrs) for Experiment 4 to mimic natural daylight at the time of our experiments. We maintained indoor testing conditions at 20°C and 30–40% relative humidity for all experiments. Maintenance food (poultry pellets including 16% protein) and water were provided *ad libitum* to all birds throughout quarantine and holding.

### Analytical chemistry

We chemically analyzed all test treatments to verify the nominal concentrations of provided and prepared SN formulations. The NWRC Analytical Chemistry Unit used ion chromatography to estimate the actual SN concentration in TSN and NESN test treatments for each of Experiments 1–3. The TSN or NESN material was dissolved in ultrapure water. An aliquot of the sample was diluted, transferred to a vial, capped, and analyzed by ion chromatography (± 0.01% and ± 0.1% SN in TSN and NESN, respectively; w/v). The method limit of detection for SN was 0.0044 μg/g. HOGGONE bait samples were analyzed by the NWRC Analytical Chemistry Unit using reversed-phase ion chromatography. A 1.0 g sample of HOGGONE bait was suspended with 10.0 mL of chloroform, extracted into water, diluted into linear range, and injected on the ion chromatograph. The calculated concentration of the diluted extract was used to determine the sample concentration. This method was validated using samples containing 1–15% nitrite. The efficiency of recovery for nitrite averaged 92% (SD = 2.4%), and the method limit of detection was 0.00036% w/w.

### Monitoring overt signs of toxicosis

Experimental investigations require that test subjects be exposed to ecologically relevant conditions (e.g., free-choice consumption experiments in the absence of environmental disturbance). Scientific ethics require that we minimize and mitigate the pain and stress of test subjects. We therefore developed our experimental protocols in collaboration with the NWRC Attending Veterinarian to meet both of these research needs. All starlings were monitored throughout the experiments to observe overt signs of SN toxicosis. Overt signs of SN toxicosis may include uncommon gaping, uncommon panting, hyporeactivity, ataxia, unconsciousness, lethargy, and lateral recumbency [25, 26]. The onset of clinical signs of SN toxicosis is variable but occurs most often within 60 minutes post-ingestion.

For Experiments 1–3, we monitored all starlings at 20-minute intervals from 0600–1000 hrs and thereafter at one-hour intervals for a minimum of eight hours subsequent to the tests. For Experiment 4, we continuously monitored all starlings exposed to SN while treated bait was present, from 0700–1200 hrs on test days 1–4, followed by two additional health checks before 1730 hrs. If we observed irreversible clinical signs of SN toxicosis (i.e., 40-minute irreversible ataxia, unconsciousness or inability to right themselves from abnormal postures) and/or excessive pain or stress during monitoring, interventional euthanasia would have been prescribed by the Study Director or their designee per American Veterinary Medical Association (AVMA) standards (e.g., CO_2_) [27]. Interventional euthanasia was never needed during these experiments.

### Experiment 1: Determination of LOAEL & NOAEL of SN formulations

**Fig 1:**
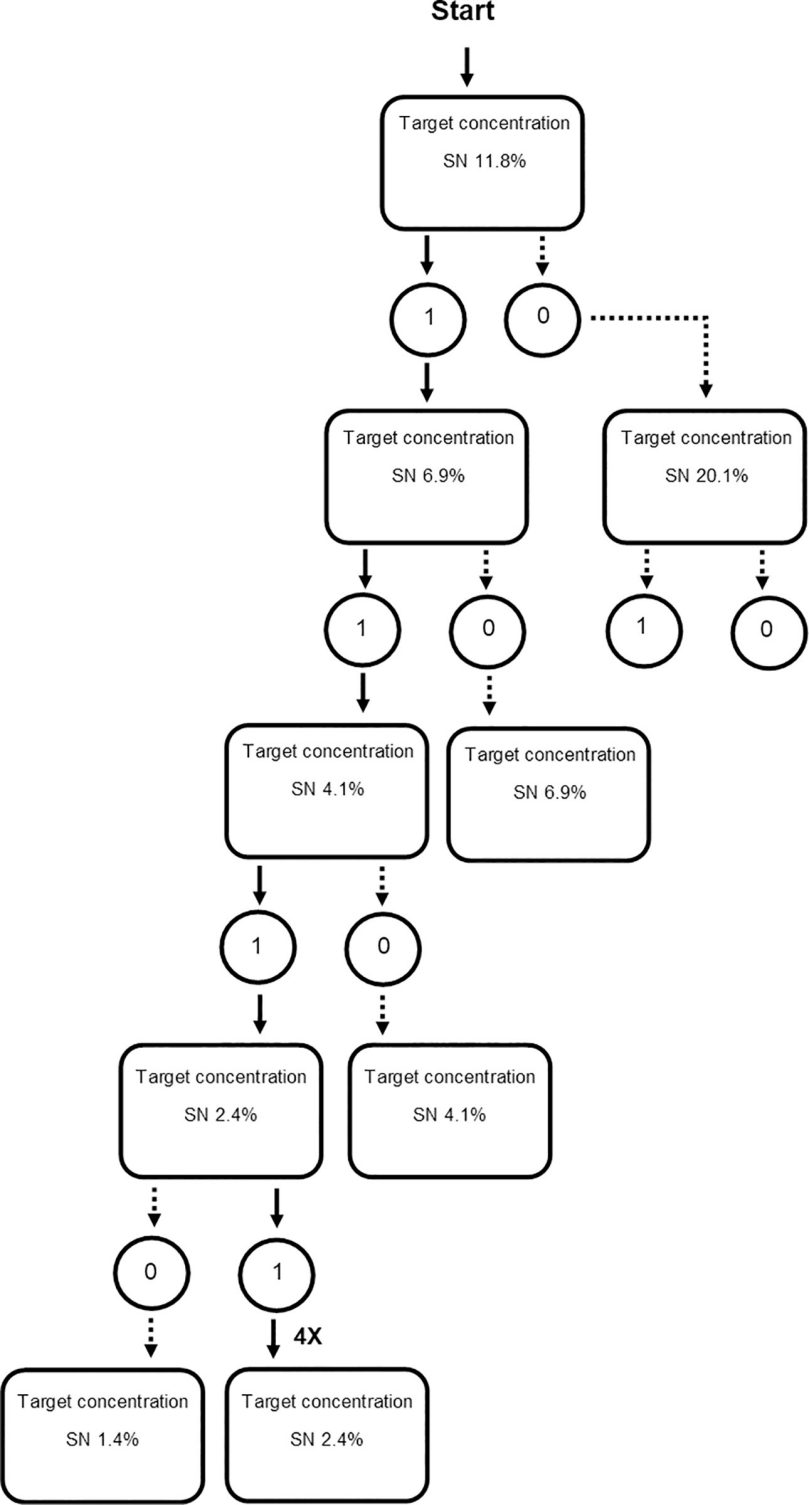
The test procedure (adapted from OCED Guideline 425) showing the process for the LOAEL/NOAEL determination for SN. This procedure uses very few animals. The acute toxicity study is a stepwise procedure, with the use of one animal per step. The acute toxicity of the test substance depends on the mortality and or moribund status of the animals, and 2–4 steps may be necessary for the evaluation. The starting dose (here 11.1% SN w/v) was tested once. If the animal died during the first test, this test was followed by testing a lower dose using a 1/1.7 progression factor. If the animal did not die during the first test, this test was followed by testing a higher dose using a 1/1.7 progression factor. Black arrows indicate the test procedure for the technical sodium nitrite group (TSN).

We used a modified up and down method (i.e., reduction in the number of utilized test subjects) to determine LOAEL and NOAEL of TSN and NESN (Fig 1) [24]. Starlings were weighed to measure pre-test body mass (BM), ranked, and assigned to one of three groups (TSN, NESN, demineralized water) such that each group was similarly populated with birds of similar BM range. We gavaged (i.e., dosed) experimentally-naïve starlings (*N* = 22) with varying aqueous concentrations of TSN and NESN to determine threshold concentrations of toxicity. Drinking water was removed from each cage at approximately 17:30 hrs on the final day of acclimation (i.e., the day prior to the test) to prepare birds for oral gavage. Gavage solutions were prepared with demineralized water, and gavage volume did not exceed 5 ml/kg BM. Ad libitum drinking water was returned to each starling at 1200 hrs. Testing was conducted on odd days of the experiment and one day of acclimation intervened between tests (i.e., ad libitum food and drinking water during even days of the experiment). Gavage solutions increased or decreased using a dose progression factor of 1.7, dependent upon toxicity or adverse effects observed during the previous test. The technical SN and nanoencapsulated SN groups progressed independently through the modified up and down method (Fig 1) until stable mortality (i.e., no mortality at the concentration tested following previously observed mortality) occurred. At which point, four starlings were gavaged at the LOAEL for their respective group. One starling from the demineralized water group was gavaged at each level of testing as a control [29]. All starlings were euthanized between 0800–1000 hrs on the day subsequent to the test.

### Experiment 2: Free-choice of LOAEL concentrations of SN formulations

Subsequent to the gavage experiment, we provided a free-choice of aqueous solutions of the TSN and NESN at LOAEL concentrations identified in Experiment 1 to determine associated toxicities in experimentally-naïve starlings (*N* = 25). For each starling, we measured daily consumption of demineralized water in each of two water containers during a 3-day pretreatment or acclimation period to determine side preferences for each test subject and to control for potential confounding of side bias during the test. Drinking water was removed from each cage at approximately 1730 hrs on the final day of acclimation (i.e., the day prior to the test).

We offered two water containers to starlings from 0600–0900 hrs on test day 1. Starlings in TSN (*n* = 10) and NESN (*n* = 10) groups were offered a free-choice of 1) approximately 75 mL of an aqueous solution of TSN or NESN, respectively, offered on the pretreatment preferred side and 2) approximately 75 mL of demineralized water offered on the non-preferred side of test cages. Starlings in the demineralized water group (*n* = 5) were offered approximately 75 mL of demineralized water in each of two water containers. We weighed (± 0.1 g) water containers offered to individual starlings prior and subsequent to the three-hour test exposure to determine individual bird water consumption. We offered approximately 75 mL of demineralized water in each of two water containers within a vacant cage to correct for changes in mass, independent of consumption (e.g., evaporation) throughout the experiment. *Ad libitum* drinking water was returned to each starling at 1200 hrs on the day of the test.

#### Statistical analysis

We used descriptive statistics ($\bar{x}$ ± SE, min, max) to summarize and t-tests (SAS 9.4; α = 0.05) to analyze starling consumption of SN-treated and untreated water during Experiment 2.

### Experiment 3: Free-choice of less than LOAEL concentrations of technical SN

We provided a free-choice of less than LOAEL concentrations of TSN identified during Experiment 2 to determine associated toxicities in experimentally-naïve starlings (*N* = 56). For each starling, we measured daily consumption of demineralized water in each of two water containers during a 3-day pretreatment, or acclimation period to determine side preferences for each test subject and control for potential confounding of side bias during the test. Both water containers were removed from each cage at approximately 1730 hrs on the final day of acclimation (i.e., the day prior to the test).

From 0600–0900 hrs on the day of the test, two water containers were offered to starlings in Groups 1–7 (*n* = 8 birds per group). Starlings in Groups 1–6 were offered a free choice of 1) approximately 75 mL of demineralized water offered on the pretreatment non-preferred side and 2) 75 mL of an aqueous solution of TSN offered on the preferred side of test cages on the single test day. Starlings in Group 7 were offered approximately 75 mL of demineralized water in each of two water containers during the single test day (untreated control). Assuming that test subjects would consume 1–15 mL of the aqueous SN solution (i.e., 1.0, 2.5, 5.0, 7.5, 10.0 or 15.0 mL for Groups 1–6, respectively) during the three-hour test, Groups 1–6 received an aqueous solution containing target concentrations of 1.85%, 0.74%, 0.37%, 0.25%, 0.19% or 0.12% of TSN (w/v), respectively. We offered approximately 75 mL of demineralized water in each of two water containers within a vacant cage throughout the experiment to correct for changes in mass independent of consumption (e.g., evaporation). Ad libitum drinking water was returned to starlings in Groups 1–7 at 1200 hrs on the day of the test.

#### Statistical analysis

We used descriptive statistics ($\bar{x}$ ± SE, min, max) to summarize and t-tests (SAS 9.4; α = 0.05) to analyze starling consumption of SN-treated and untreated water during Experiment 3.

### Experiment 4: Acute dietary toxicity test

Because starlings sometimes occupy sites associated with toxic bait applications for the control of invasive wild pigs and starlings could serve as a model for native bird species, we conducted a fourth experiment to determine if the SN formulated into HOGGONE bait represented a non-target hazard to starlings. Experiment 4 was a dietary toxicity test with two groups of experimentally-naïve starlings. Starlings acclimated within each of two, visually-isolated group cages for five days (*n* = 10 birds per cage). During the acclimation period, one food bowl containing 1.5 kg of the maintenance diet was presented at 0800 hrs daily for four days. We removed the maintenance diet after 24 hours and quantified consumption (± 0.1g). We placed 1.5 kg of the maintenance diet outside of the group cage throughout the experiment to correct for changes in mass, independent of consumption (e.g., desiccation). Starlings had access to water *ad libitum* throughout the experiment. The maintenance diet was removed at approximately 1730 hrs on the final day of acclimation (i.e., the day prior to the test).

The four-day test immediately followed the acclimation period. One food bowl containing 1.0 kg HOGGONE paste bait (10% SN or 0% SN w/w) was presented at 0700 hrs daily for four days. The paste bait was broken up in the bowl and presented in pieces (approx. 5 cm^3^). HOGGONE bait was removed at 1200 hrs and weighed daily to quantify consumption. One food bowl containing 1.5 kg of the maintenance diet was presented within each cage at 1200 daily for four days. The maintenance diet was removed at approximately 1730 hrs for four days, and test consumption (including desiccation) was quantified daily. Starlings were euthanized on the day following the last day of the test.

#### Statistical analysis

We used descriptive statistics ($\bar{x}$ ± SE, min, max) to describe consumption of the maintenance diet and SN-treated and untreated HOGGONE during Experiment 4.

## Results

### Experiment 1: Determination of LOAEL & NOAEL of SN formulations

We observed 100% mortality (i.e., one of one test subjects) in the TSN group at our targeted concentrations of 11.8% (ion chromatographic actual concentration = 13.0%), 6.9% (7.36%), 4.1% (4.10%), and 2.4% (2.40%) SN (w/v), respectively (Group 1, Table 1). The time-to-death for individual starlings was 29, 31, 22, and 47 minutes, respectively. We observed 0% mortality in the TSN group at 1.4% (1.3%) SN (w/v) (Table 1). The time-to-death for the TSN group at the LOAEL concentration ranged from 34–63 minutes. The LOAEL for mortality of the TSN was 2.40% SN (actual concentration, or 120 mg/kg) and the NOAEL for mortality of the TSN was 1.30% SN (actual concentration, or 65 mg/kg; Table 1) during Experiment 1.

**Table 1:** Test-related mortalities among European starlings gavaged (i.e., dosed) with technical sodium nitrite (TSN), nanoencapsulated sodium nitrite (NESN), or demineralized water (untreated control) in Experiment 1.

		TSN		NESN		Water
Test (*n*)	Target dose [SN] %	Actual dose [SN] %	Test-related Mortalities	Actual dose [SN] %	Test-related Mortalities	Test-related Mortalities
1(1)	11.8	13.0	1/1	5.8	1/1	0/1
2(1)	6.9	7.4	1/1	3.5	1/1 (LOAEL)	0/1
3(1)	4.1	4.1	1/1	1.7	0/1 (NOAEL)	0/1
4(1)	2.4	2.4	1/1 (LOAEL)	-	-	0/1
5(1)	1.4	1.3	0/1 (NOAEL)	-	-	0/1
6(4)	2.4 (LOAEL)	2.4	3/4	-	-	
6(4)	6.9 (LOAEL)	-	-	3.3	3/4	
6(1)	0					0/1

Comparative toxicity was used to determine the lowest observed adverse effect level (LOAEL) and no observed adverse effect level (NOAEL) among various sodium nitrite concentrations ([SN]) in European starlings.

We observed 100% mortality (i.e., one of one test subjects) in the NESN group at manufacturer-recommended target concentrations of 11.8% (ion chromatographic actual concentration = 5.84%) and 6.9% (3.46%) SN (w/v), respectively (Table 1). The time-to-death for individual starlings were 24 and 26 minutes, respectively. We observed 0% mortality in the NESN group at 4.1% (1.7%) SN (w/v) (Table 1). The time-to-death for the NESN group at the LOAEL concentration ranged from 18–70 minutes. The LOAEL for mortality of NESN was 3.46% SN (actual concentration, or 173 mg/kg) and the NOAEL for mortality of NESN was 1.74% SN (actual concentration, or 87 mg/kg; Table 1) during the gavage experiment. As expected, we observed no mortalities among starlings gavaged with demineralized water during the gavage experiment (Table 1).

Although the target concentration of the NESN was expected to be 25% SN (w/v), the actual SN concentration was 12.1% (SEM = 0.09%). Actual concentrations of SN in all untreated controls was less than the method limit of detection associated with ion chromatographic analyses (MLOD = 0.0044 μg SN/g).

### Experiment 2: Free-choice of LOAEL concentrations of SN formulations

We observed 40% mortality (i.e., four of 10 test subjects) among the TSN group offered the LOAEL concentration (2.4% SN) of demineralized water treated with TSN (Table 2). Consumption of demineralized water treated with TSN was less than that of untreated water during this free-choice experiment (t9 = ˗2.22, P = 0.0537). Average consumption of the aqueous solution of TSN was 1.6 mL (± 0.1 SE; min, max = 1.1, 2.6 mL) and average consumption of demineralized water was 3.9 mL (± 1.0; min, max = 0.7, 9.0 mL) during Experiment 2.

**Table 2:** Test-related mortalities among European starlings offered a free-choice of demineralized water treated with technical sodium nitrite (TSN) or AquaPower nanoencapsulated sodium nitrite (NESN) and demineralized water in Experiment 2.

			Test-related Mortalities		
Group (*n*)	Target[SN] %	Actual[SN] %	TSN	NESN	Water
TSN (10)	2.40 (LOAEL)	2.44	4/10		
NESN (10)	6.9 (LOAEL)	3.39		2/10	
Water (5)	0	<MLOD			0/5

The demineralized water group (untreated control) received two water containers of demineralized water during the test. Test treatments were based upon the lowest observed adverse effect levels (LOAEL) or sodium nitrite concentration ([SN]) identified during Experiment 1. The method limit of detection (MLOD) associated with ion chromatographic analyses was MLOD = 0.0044 μg SN/g.

We observed 20% mortality (i.e., two of 10 test subjects) among starlings offered the LOAEL concentration (6.9% SN) of water treated with the NESN (Table 2). Consumption of demineralized water treated with the NESN was less than that of untreated water (t9 = ˗3.18, P = 0.0112) during this free-choice experiment. Average consumption of the water treated with the NESN was 1.4 mL (± 0.1 SE; min, max = 1.0, 2.2 mL) and average consumption of demineralized water was 9.2 mL (± 2.5; min, max = 0.4, 24.9 mL) during Experiment 2. As expected, we observed zero moralities among starlings offered two water containers of demineralized water (i.e., untreated controls; Table 2).

### Experiment 3: Free-choice of less than LOAEL concentrations of technical SN

**Fig 2:**
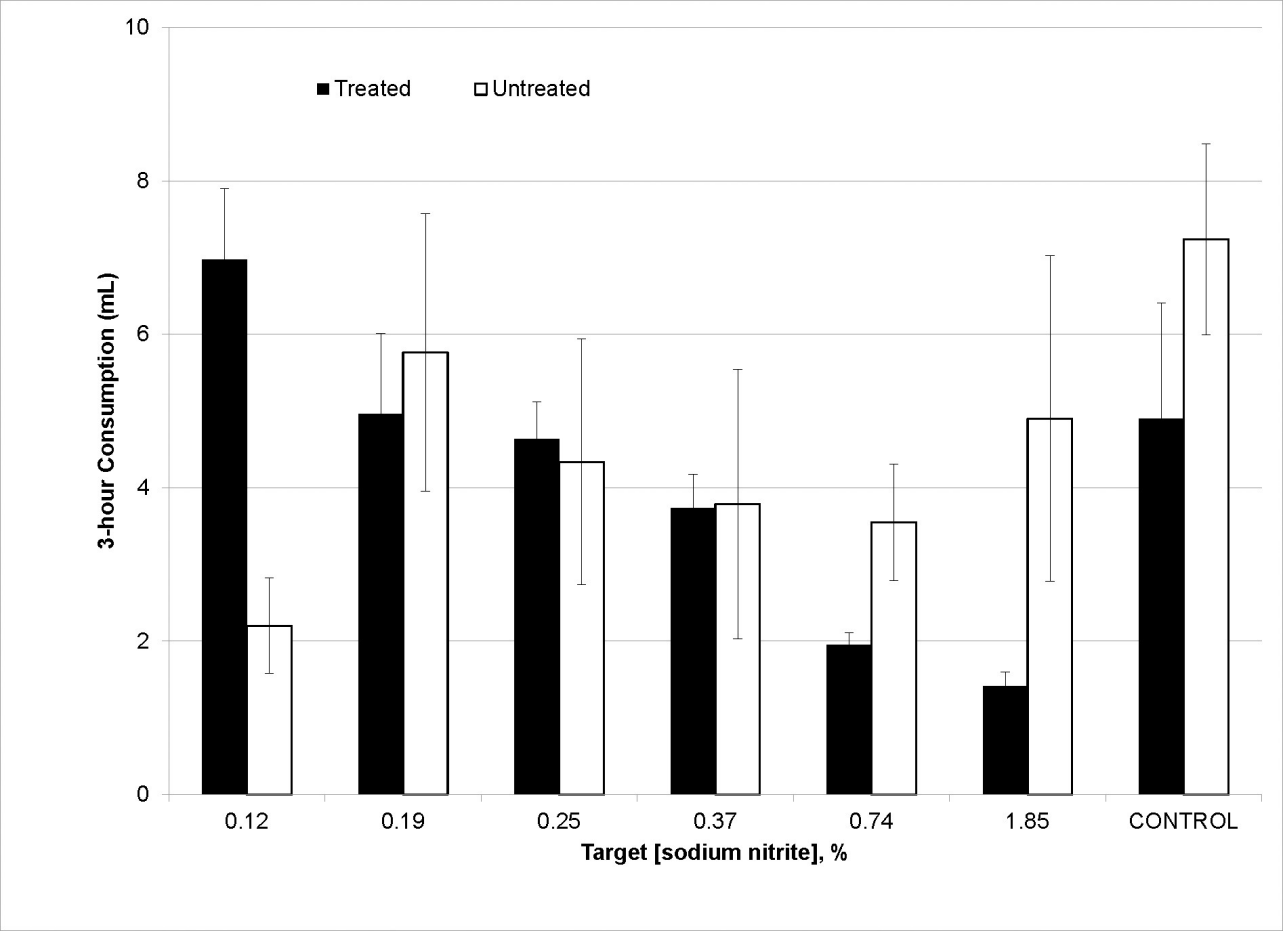
Average consumption (± SE) of demineralized water treated with technical sodium nitrite and untreated water in Experiment 3. This experiment was designed to determine associated toxicities in experimentally-naïve European starlings.

Starlings in Groups 1–6 received a free-choice of demineralized water and water treated with TSN. We observed 12.5% mortality (i.e., one of eight test subjects) among starlings in Group 1 (Table 3). For Group 1, consumption of demineralized water treated with 1.85% TSN (w/v) (ion chromatographic actual concentration = 1.88%) was similar to that of untreated water (t7 = ˗1.56, P = 0.1636) during this free-choice experiment. Average consumption of the aqueous solution of 1.85% TSN was 1.4 mL (± 0.2 SE; min, max = 0.6, 2.5 mL) and average consumption of demineralized water was 4.9 mL (± 2.1; min, max = 0.7, 19.1 mL; Fig 2).

**Table 3:** Test-related mortalities among European starlings offered a free-choice of demineralized water treated with technical sodium nitrite (SN) and demineralized water in Experiment 3.

			**Test-related Mortalities by Group**						
**Group (*n*)**	**Target [SN] (%)**	**Actual [SN] (%)**	**1**	**2**	**3**	**4**	**5**	**6**	**7**
1 (8)	1.85	1.880	1/8						
2 (8)	0.74	0.750		0/8					
3 (8)	0.37	0.363			0/8				
4 (8)	0.25	0.248				0/8			
5 (8)	0.19	0.188					0/8		
6 (8)	0.12	0.127						0/8	
7 (8)	0	<MLOD							0/8

The method limit of detection (MLOD) associated with ion chromatographic analyses was 0.0044 μg SN/g.

We observed zero mortalities among starlings in each of Groups 2–7 during this free-choice experiment. For Group 6, consumption of demineralized water treated with 0.127% TSN (w/v) was greater than that of untreated water during this free-choice experiment (t7 = 4.50, P = 0.0028). We observed no statistical differences for the consumption of SN-treated and untreated water among starlings in each of Groups 2–5 and Group 7 (untreated control) (Fig 2).

### Experiment 4: Acute dietary toxicity test

**Fig 3:**
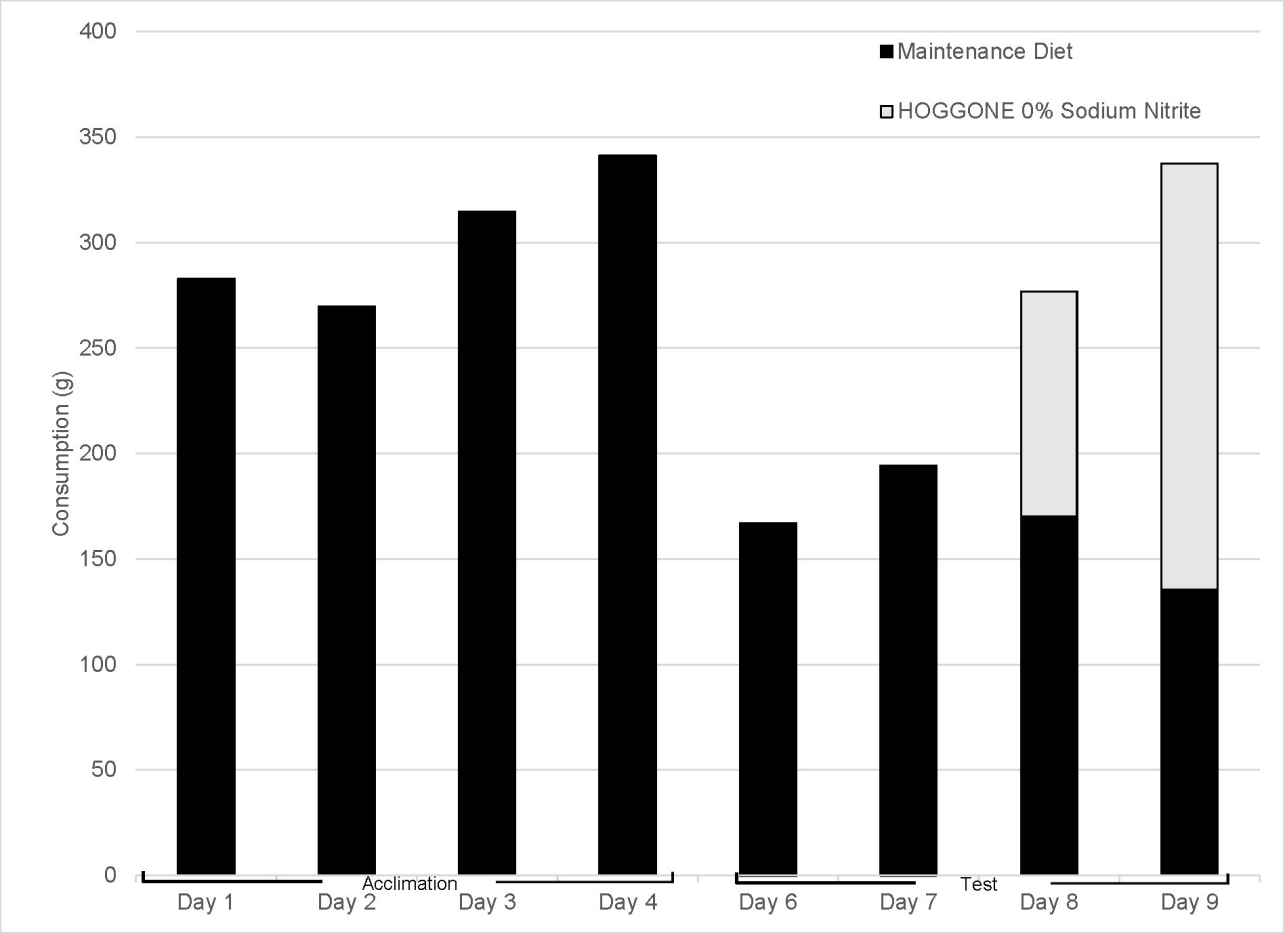
Consumption of the maintenance diet and untreated HOGGONE bait (0% sodium nitrite) in Experiment 4. This experiment was designed to determine how experimentally-naïve European starlings interact with the novel HOGGONE bait matrix.

**Fig 4:**
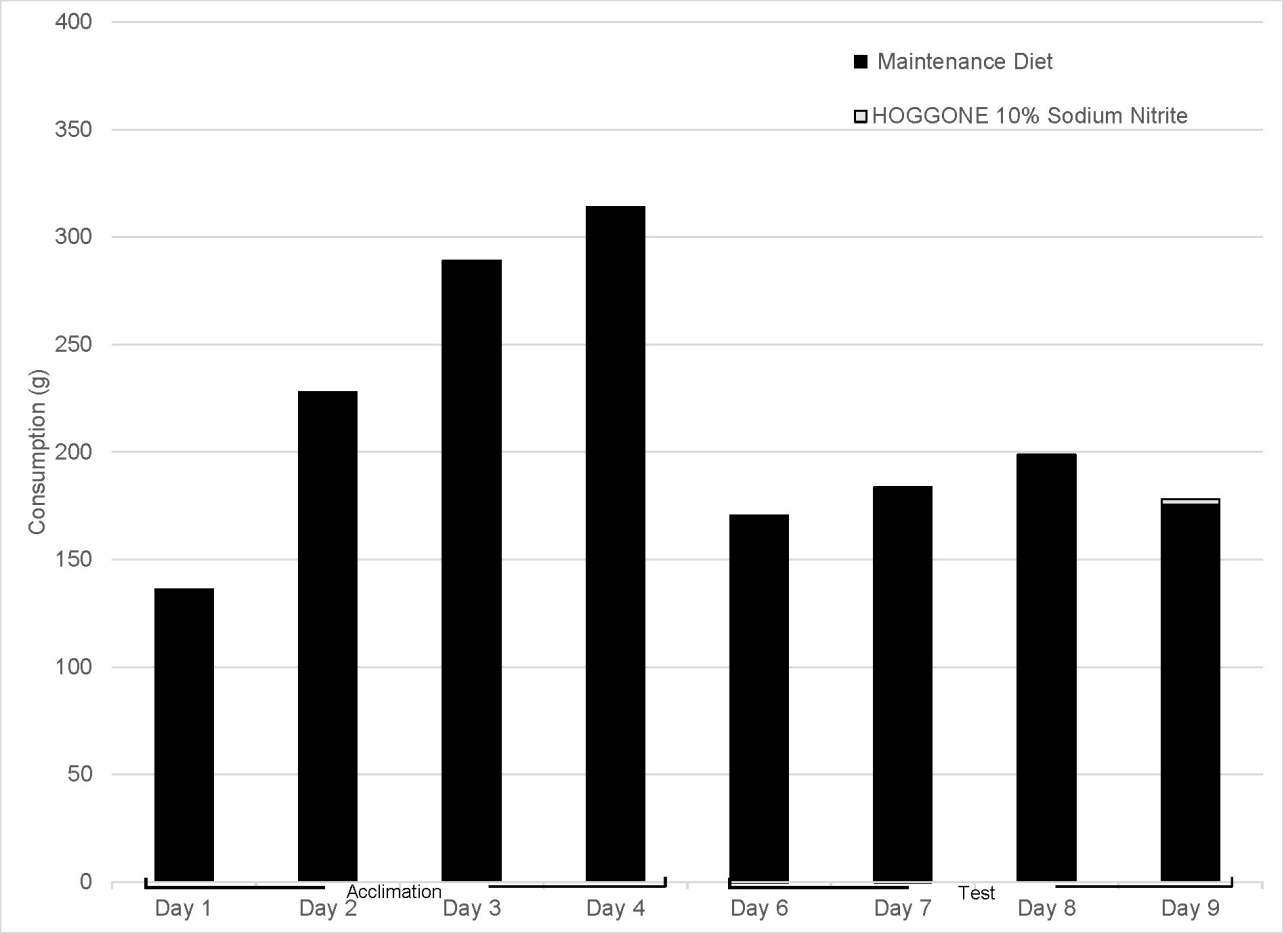
Consumption of the maintenance diet and treated HOGGONE bait (10% sodium nitrite) in Experiment 4. This experiment was designed to determine how experimentally-naïve European starlings interact with the novel HOGGONE bait matrix and identify associated mortality or morbidity.

During days one and two of the four-day test, neither group of starlings consumed a measurable quantity of HOGGONE bait. On days three and four of testing, the 0% SN HOGGONE group consumed 106.7 g and 201.9 g of 0% SN HOGGONE, respectively (Fig 3). In contrast, the 10% SN HOGGONE group consumed 0.3 and 3.8 g of 10% SN HOGGONE on days three and four, respectively (Fig 4). We observed one mortality in the 10% SN HOGGONE group on day four following observed consumption of HOGGONE bait. Time-to-death for this individual was approximately 45 minutes. The SN concentration (% w/w) in HOGGONE paste bait was 10.1% (SE = 0.33%). Daily consumption varied among test groups in Experiment 4 (Table 4).

**Table 4:** Consumption (g) of the maintenance diet and the HOGGONE bait (0% and 10% sodium nitrite) among European starlings during the acclimation and test phases of Experiment 4.

	Consumption					
	Acclimation		Test			
Group	Maintenance Diet	Std. Error	Maintenance Diet	Std. Error	HOGGONE	Std. Error
0% SN	302.2	16.1	166.7	12.0	77.0	48.7
10% SN	241.8	36.9	182.0	6.1	0.6	0.8

Starlings were euthanized, and final BM was measured (g) at the conclusion of the fourth day of testing. The Veterinary Diagnostic Laboratory at Colorado State University (Fort Collins, CO, USA) necropsied the single mortality from the test group exposed to SN. Gross necropsy showed slightly darkened lungs and blood accumulation, consistent with SN poisoning. All other internal organs were within normal limits.

## Discussion

Technical SN and nanoencapsulated SN have moderate potential as a European starling toxicant. The oral gavage LOAEL 120–173 mg/kg for TSN and NESN in Experiment 1 was similar to values reported for blackbirds and represented a moderate hazard to starlings for SN toxicity in liquid form. We conducted Experiment 1 with single subjects until we identified the LOAEL of SN in starlings. We then replicated our gavage experiment with four additional starlings offered 2.4% SN. Although our inferences are constrained by our low sample size used in Experiment 1, we used only 22 starlings to evaluate test-related mortalities associated with five concentrations of TSN and three concentrations of NESN. We then proceeded with well-replicated Experiments 2 & 3 (*n* = 8–10 starlings per group offered TSN and NESN). Oral gavage LD_50_ values for SN in solution range from 68.50 mg/kg for chickens and ducks, 120 mg/kg in blackbirds (*Turdus merula* L.), 588 mg/kg in domestic turkeys (*Meleagris gallopavo* L.), to 619 mg/kg in bobwhite quail (*Colinus virginianus* L.) [28]. Previous research suggested an LD_50_ less than 100 mg/kg as the effective threshold for successful avian toxicants [29]. Bird species with LD_50_s greater than 150 mg/kg (e.g., DRC-1339, 3-chloro-4-methylaniline hydrochloride; CAS number 7745-89-3) are considered moderately tolerant [30].

During the first free-choice drinking experiment (Experiment 2), the consumption of TSN- and NESN-treated water was similar at respective LOAELs. Still, mortality differed with higher observed mortality in the TSN group (40% mortality) than in the NESN group (20% mortality). It has been hypothesized that birds use ultraviolet (UV) wavelengths for detecting food that either absorbs or reflects strongly in the UV relative to the background [31]. Sodium nitrite absorbs UV light between 340–360 nm, potentially making both TSN and NESN treatments visible to birds that are UV sensitive, such as starlings [32]. The relatively low level of observed mortality for the water treated with NESN was unexpected. We are unsure if the nanoencapsulation of SN reduced the absorption of SN by starlings, thus allowing birds to consume similar amounts of treated water with lower observed mortality because the NESN passed through their digestive system. This result cannot be confirmed with the data from the current study because the nanoencapsulation was proprietary. We are, therefore, unsure of the mechanism for its release of SN.

Our second free-choice drinking experiment (Experiment 3) was conducted to determine if birds would consume sufficient quantities of SN treatments at concentrations lower than the LOAEL to cause SN toxicosis. Even at concentrations below the LOAEL, consumption of SN-treated water was typically lower than the consumption of untreated water. One exception (i.e., one starling) occurred at the lowest concentration in which SN-treated water consumption was greater than the consumption of untreated water but with no observed mortalities. This consumption might suggest that, at 1.88% SN, most starlings were able to detect the presence of SN in solution either by taste or visual cue and avoided ingesting a lethal dose. Sodium nitrite is a heavy salt, and there have been palatability issues associated with developing SN as a toxicant for wild pigs. Thus, researchers have resorted to microencapsulating the SN to improve consumption of toxic bait [17, 19]. An alternate possibility is that, at relatively low SN concentrations (e.g., ≤ 0.75% SN), starlings may have been able to process the SN via an efficient ascorbate reductase system or low intracellular anion concentration and thus avert toxic effects [33]. The future development of SN as an avian toxicant is dependent upon its cost-effectiveness. We, therefore, discontinued testing of SN as a primary toxicant for starlings following Experiments 1–3.

Though TSN and NESN have moderate potential as a starling toxicant in solution, we conducted Experiment 4 to evaluate the level of non-target hazard that HOGGONE may pose to starlings and other avifauna. The experimental HOGGONE bait contained 100 mg SN per 1.0 g of bait [21]. Based upon the oral gavage LOAEL for TSN and NESN, starlings would need to consume 0.10–0.14 g of bait to show adverse effects from the consumption of SN. We were not able to determine individual SN ingestion during our free-choice and acute dietary toxicity experiments. If we assume that all ten test birds ate equal amounts of the formulated bait, then each starling would have consumed 0.28 g on test day 4. Based upon our LOAEL calculations, this is sufficient to have caused mortality in all ten starlings. However, only one mortality was observed in Experiment 4. Previous testing with chickens showed an increased LD50 for dietary toxicity compared to gavage toxicity (254.6 mg/kg compared to 68.5 mg/kg) [28]. Similar differences in toxicity have been observed in brushtailed possums (*Trichosurus vulpecula*) offered sodium monofluoroacetate baits vs. water [34]. This difference in toxicity of formulated baits vs. water solutions may also occur in European starlings. We were unable to calculate an LD50 for dietary toxicity based upon our data.

Shapiro et al. [28] also conducted gavage and free-feeding trials with SN in live birds, including chickens (*Gallus gallus domesticus* L.), domestic mallard ducks (*Anas platyrhynchos domesitca* L.), pigeons (*Columba livia f*. *domestica* Gmelin), and budgerigars (*Melopsittacus undulatus* Shaw). During all free-feeding trials, Shapiro et al. [28] offered birds non-toxic bait (paste or pellet form) for one week prior to the four-hour (i.e., single day) exposure to toxic baits to mimic pre-baiting of sites during actual vertebrate pest control. We chose to offer the test matrix (paste bait) only during the four-day test (five hours/day). Our test design is similar to the protocol for EPA Ecological Effects Test Guidelines OCSPP 850.2200: Avian Dietary Toxicity Test (EPA 812-C-024 January 2012). Our methodology also mimicked how birds may encounter bait in the field in the US, where it would be offered within bait stations that are species-specifically designed for wild pigs.

Birds in the wild could habituate to wild pig bait sites during the initial phase of baiting (i.e., grain baiting on the ground), prior to the introduction of bait stations. Wild pigs have spilled small quantities of toxic paste crumbles near bait stations during field applications of HOGGONE. Exposure of birds to the toxic bait would most likely be through this spillage of the toxic bait by wild pigs. Non-target birds would therefore have limited access to and experience with the toxic bait. Our testing showed that starlings in both untreated and SN-treated groups were slow to accept the paste bait as a food source. Birds in the untreated group consumed amounts of the paste bait equivalent to the maintenance diet on days three and four. In contrast, birds in the SN-treated group continued to avoid the paste bait, and they consumed only minuscule amounts of the treated bait in 4 days of testing. Birds that encounter toxic HOGGONE in the field may not be enticed to consume the bait if other foods are readily available or weather conditions do not necessitate high caloric uptake. Yet to be published data from field trials focused on wild pigs, though, suggest that birds can be attracted to crumbs of paste bait spilled by wild pigs and that this spillage can be toxic to birds (K. VerCauteren, personal observation).

Novelty plays an important role in the process of food selection [35]. In our experience, starlings are reluctant to sample novel foods, especially when birds were individually caged. Caging starlings in small groups (e.g., 5–10 birds) can facilitate the sampling of novel foods. However, the starling’s initial reluctance to consume the paste bait in this study could also be related to the bait’s visual cues (e.g., black coloration).

We conducted all testing for this study in indoor testing facilities, and we maintained room temperatures at 20°C. When ambient temperatures are below the lower critical temperature (LCT; the temperature where heat production must increase or heat loss must decrease), starlings and other birds increase their metabolic rate to compensate for heat loss (i.e., thermoregulation). Increases in metabolic rate cause a concomitant increase in food demand [36]. A study of rock pigeons (*Columba livia*) and house sparrows (*Passer domesticus*) described increased food consumption during winter months as compared to summer [37]. Therefore, had our study been conducted at lower ambient temperatures, starlings may have been motivated to consume the bait earlier than days 3–4. Future research with HOGGONE would benefit from testing at temperatures that would necessitate a higher food demand and thus approximate seasonal field conditions associated with toxic bait applications for the management of wild pig damages.

## Conclusion

Based on our cage testing, SN toxicity presented a moderate hazard to European starlings. Starlings are moderately sensitive to SN poisoning (LOAEL 120–170 mg/kg) in oral gavage studies, but the LOAEL of SN for birds should be determined under field conditions (e.g., 1.3–2.4% SN offered to free-ranging starlings in a controlled field experiment). In our captive experiments, pre-ingestive detectability presented a potential problem for SN as an avian toxicant. Conversely, pre-ingestive detection may be used to reduce non-target hazards of SN for birds associated with wild pig damage management. Future studies are recommended with other North American passerine species to determine the non-target risk of SN toxicity at bait sites and to limit the availability of SN bait to birds due to spillage by feeding wild pigs. These risks could be minimized by following best practices for vertebrate pest baiting. These practices could include species-specific bait stations with a non-lethal frightening device or non-target birds. The frightening device would operate from 1-hr before sunrise the morning after deploying toxic bait (i.e., after potential wild pig spillage occurs) until personnel arrives at the site to remove the toxic bait.
